# A *T*elehealth-supported, *I*ntegrated care with CHWs, and *ME*dication-access (TIME) Program for Diabetes Improves HbA1c: a Randomized Clinical Trial

**DOI:** 10.1007/s11606-020-06017-4

**Published:** 2020-07-22

**Authors:** Elizabeth M. Vaughan, David J. Hyman, Aanand D. Naik, Susan L. Samson, Javad Razjouyan, John P. Foreyt

**Affiliations:** 1 Division of General Internal Medicine, Department of Medicine , Baylor College of Medicine, Houston, TX USA; 2 Center for Innovations in Quality, Effectiveness, and Safety (IQuESt), Michael E DeBakey VA Medical Center, Houston, TX USA; 3 Division of Health Services Research, Department of Medicine, Baylor College of Medicine, Houston, TX USA; 4 Division of Geriatrics and Palliative Medicine, Department of Medicine, Baylor College of Medicine, Houston, TX USA; 5 Section of Endocrinology, Diabetes and Metabolism, Department of Medicine, Baylor College of Medicine, Houston, USA

**Keywords:** diabetes, medication, low-income, group visits or shared medical appointments, community health worker, obesity

## Abstract

**Background:**

Many individuals with diabetes live in low- or middle-income settings. Glycemic control is challenging, particularly in resource-limited areas that face numerous healthcare barriers.

**Objective:**

To compare HbA1c outcomes for individuals randomized to TIME, a *T*elehealth-supported, *I*ntegrated care with CHWs (Community Health Workers), and *ME*dication-access program (intervention) versus usual care (wait-list control).

**Design:**

Randomized clinical trial.

**Participants:**

Low-income Latino(a) adults with type 2 diabetes.

**Interventions:**

TIME consisted of (1) CHW-participant telehealth communication via mobile health (mHealth) for 12 months, (2) CHW-led monthly group visits for 6 months, and (3) weekly CHW-physician diabetes training and support via telehealth (video conferencing).

**Main Measures:**

Investigators compared TIME versus control participant baseline to month 6 changes of HbA1c (primary outcome), blood pressure, body mass index (BMI), weight, and adherence to seven American Diabetes Association (ADA) standards of care. CHW assistance in identifying barriers to healthcare in the intervention group were measured at the end of mHealth communication (12 months).

**Key Results:**

A total of 89 individuals participated. TIME individuals compared to control participants had significant HbA1c decreases (9.02 to 7.59% (− 1.43%) vs. 8.71 to 8.26% (− 0.45%), respectively, *p* = 0.002), blood pressure changes (systolic: − 6.89 mmHg vs. 0.03 mmHg, *p* = 0.023; diastolic: − 3.36 mmHg vs. 0.2 mmHg, respectively, *p* = 0.046), and ADA guideline adherence (*p* < 0.001) from baseline to month 6. At month 6, more TIME than control participants achieved > 0.50% HbA1c reductions (88.57% vs. 43.75%, *p* < 0.001). BMI and weight changes were not significant between groups. Many (54.6%) TIME participants experienced > 1 barrier to care, of whom 91.7% had medication issues. CHWs identified the majority (87.5%) of barriers.

**Conclusions:**

TIME participants resulted in improved outcomes including HbA1c. CHWs are uniquely positioned to identify barriers to care particularly related to medications that may have gone unrecognized otherwise. Larger trials are needed to determine the scalability and sustainability of the intervention.

**Clinical Trial:**

NCT03394456, accessed at https://clinicaltrials.gov/ct2/show/NCT03394456

**Electronic supplementary material:**

The online version of this article (10.1007/s11606-020-06017-4) contains supplementary material, which is available to authorized users.

## INTRODUCTION

Eighty percent of individuals with diabetes live in low- or middle-income countries.^1^ In high-income countries, most (69%) of those suffering from diabetes are low- or middle-income.^2^ Resource-limited and minority populations have a greater prevalence of diabetes and more disease-related complications.^1^ Strategies to improve glycemic control are complicated by poverty, limited literacy, and access to medications and healthcare.^3, 4^ These severe disparities highlight the need for innovative changes.

One strategy to improve care in resource-limited settings is community health workers (CHWs). These individuals have been instrumental in improving patient outcomes for a variety of diseases and conditions including diabetes.^5–8^ Our recent study showed that CHWs successfully led diabetes group visits.^6^ Group visits or shared medical appointments are a cost-effective intervention, comprising of education, integrated primary care (e.g., medication reconciliation), and goal development.^9–11^ Most group visits are led by physicians or other health professionals^12^ and, though bilingual providers add value in relating to the patient population, they are in short supply.^13^ Integrating CHWs into group visits has showed value in overcoming cultural and language barriers.^6^

However, there have been major concerns related to inadequate CHW training and support.^7^ This has led to increased global interest in telehealth mechanisms including mobile health (mHealth), particularly as they relate to supporting CHWs by assisting with diagnostics, enhancing communication, and professional development, and, thereby, improving patient care.^5, 14, 15^ A study of 72 African American individuals revealed that CHW-participant mHealth collaboration improved participant diabetes self-management skills and provided support by connecting CHWs to the healthcare team.^14^ A systematic review and meta-analysis illustrated numerous benefits of CHW-participant mHealth interaction including appointment reminders and reporting from peripheral sites.^5^

Though prior studies have demonstrated the effectiveness of CHWs and mHealth,^5, 8, 14^ their combination has not been evaluated. To evaluate this combined strategy in a clinical trial, investigators compared individuals with type 2 diabetes randomized to the program TIME (*T*elehealth-supported, *I*ntegrated care with CHWs, and *ME*dication-access) (intervention) versus usual care in the clinic (control). TIME consisted of CHW-participant mHealth communication, CHW-led diabetes group visits, and CHW-physician diabetes training and support via telehealth (video conferencing). Investigators evaluated clinical outcomes (i.e., HbA1c (primary), adherence to American Diabetes Association (ADA) standards of care^3^) and CHW assistance in identifying barriers to healthcare. The overarching hypothesis was improved glycemic control of TIME compared to control participants.

## METHODS

### Study Design and Setting

This was a randomized, wait-list clinical trial conducted at a nonprofit clinic in Houston, Texas. Clinic eligibility included uninsured individuals earning < 250% of the federal poverty level. The Institutional Review Board at Baylor College of Medicine approved the study. Written consent and signed group visit confidentiality forms were obtained from study participants.

### Identification, Recruitment, and Retention of Participants

We recruited two cohorts 6 months apart. Eligible participants were adults (> 18 years) with type 2 diabetes (HbA1c > 6.5%), self-identified as Latino(a), and Spanish-speaking. Individuals were excluded if they were pregnant, had an event that would alter HbA1c levels (e.g., recent blood transfusion), or did not attend at least one group visit. Methods to identify potential participants were through the clinic database (primary), clinic referrals, or word of mouth. The clinic database filtered for Latino(a)/Hispanic with diabetes (ICD-10 E11:9, E11:X). Study staff contacted eligible individuals by phone to explain the study and interested individuals were invited to an orientation. At orientation, investigators obtained baseline clinical and survey data, signed written consent, and randomized participants to TIME (intervention) or usual care (wait-list control) using an automatic number generator to achieve block randomization. A physician completed a secondary chart review to confirm eligibility.

To address the issue of participant retention in low-income settings,^10^ TIME included several strategies including establishing an efficient tracking system, CHW training to identify participant barriers, and room setup with intentional socialization periods arranged to promote a welcoming environment.^16^ Meals, childcare, in-class laboratory, and medication refills also were provided.^16^

### Clinical Intervention Program

TIME consisted of CHW-participant mHealth communication, CHW-led diabetes group visits, and CHW-physician diabetes training and support via telehealth. Usual care in the clinic included diabetes management with physicians (quarterly) and clinical pharmacists (monthly) in addition to routinely offered nutrition classes. To allow wait-list control individuals the opportunity to receive TIME,^17^ they were offered the intervention months 7–12. After receiving the group visits, participants returned to usual care. Month 6 HbA1c, blood pressure, weight, and survey data were gathered for the wait-list control group prior to them receiving the intervention. Attrition was defined as study staff inability to contact a participant by month 6.

#### Community Health Workers

CHWs (*n* = 6) were self-identified as Latino(a), fluent in Spanish, and maintained their state of Texas CHW certification (e.g., initial: 160-h certification course; renewal: 20-h continuing education biennially).^18^

#### *T*elehealth-support

In our feasibility study, CHWs received quarterly, in-person training^6^ while in this investigation, they attended weekly video conferencing (ZOOM^19^) meetings, which has been published elsewhere.^15^ In summary, a study physician met with CHWs for weekly training that paralleled the monthly group visit topics (30 min) and support (30 min) to address questions or concerns. This telehealth venue was well-accepted and significantly improved CHWs’ diabetes knowledge (*p* < 0.001).^15^

A second telehealth support mechanism for CHWs and participants was mHealth. Each CHW was assigned 3–4 participants of whom they contacted weekly (months 1–6) or bimonthly (months 7–12) (call or text pending participant preference) to inquire about glucose control, medication adherence, and questions or concerns. CHWs taught their participants how to use a secure, encrypted app (OhMD^20^) for communication. Control participants followed this same pattern but it was delayed 6 months. They did not have any CHW contact while in usual care (months 1–6).

#### *I*ntegrated care with CHWs

We utilized our feasibility study for the group visit structure and curriculum (Table 1).^6^ Briefly, group visits were in Spanish, met monthly for 6 months on Saturdays from 9A-12P, and had the following format: vital signs, large group education, three small groups (*n* = 30 min/section), and a healthy meal. The three small groups addressed medical, social, and behavioral barriers to care. The large group topics focused on diabetes self-management and standards of care.^3, 21–23^ Participants briefly met with a bilingual study physician individually for medication management. CHWs led the large group and social and behavioral small groups. Materials were available in English and in Spanish.

**Table 1:** Monthly Group Visit Activities and Curriculum for the TIME (*T*elehealth-supported, *I*ntegrated care with Community Health Workers, and *ME*dication-access) Program

Time (min)	Activity
30	Vitals, labs (if indicated)
30	Large group educational session
90 (30/group)	Three small groups: medical management; social barriers to care; psychological barriers to care
30	Healthy meal and conclusions
Total: 3 hours per group visit	
Curriculum during large group^21^ (chapter)	
Month 1: diabetes overview (1, 2, 18)	
Month 2: medication adherence (13)	
Month 3: nutrition (11, 12)	
Month 4: prevention (chapters 13, 19)	
Month 5: sex, depression, and diabetes (4, 5, 10)	
Month 6: physical activity (6–8)	

#### *ME*dication-access

To address challenges of adherence in low-income settings,^24^ a study physician prescribed medications offered at low costs: metformin, glimepiride, and pioglitazone. The latter two were chosen as they are associated with fewer adverse events than similar drugs from the same class.^25, 26^

### Study Outcomes

#### Clinical

The primary study outcome was glycemic control as measured by baseline to month 6 change of HbA1c. To evaluate individuals who maintained or achieved target (e.g., < 7.0%)^3^ by month 6 and assess the number who achieved clinically meaningful decreases in HbA1c (> 0.5%)^27^, investigators also conducted a sub-analysis named *responders*, defined as meeting either or both markers. Other clinical outcomes were blood pressure, weight, and BMI, of which investigators obtained at baseline (orientation) and 6 months (month 6 group visits). Baseline to month 6 adherence to seven ADA standard of care markers were collected through chart review at the following timepoints: baseline/month 6 (appropriately dosed statin, B12 screening), baseline/month 6 and 1 year prior to each timepoint (annual screenings: retinal, comprehensive foot exam, microalbuminuria, influenza vaccination), and baseline/month 6 and time prior to each timepoint as dictated per current recommendations (pneumococcal vaccination).^3^

Clinical outcomes were measured after the completion of the diabetes group visits rather than at the end of CHW-participant contact (12 months) due to introduction of multiple variables upon return to clinic including potential changes of providers, eligibility, and clinics. Month 6 was defined as 24 weeks after orientation ± 4 weeks. To reduce bias toward the intervention group who may have had more frequent checks, account for potential time variations between study arms, and collect data of participants not present at group visits, a study physician conducted a secondary chart review for all participants and recorded the lowest month 6 clinical value for HbA1c, blood pressure, and weight. Study team technique for obtaining clinical measures reflected those of the clinic (e.g., recheck blood pressure in both arms after 15 min for levels > 140/90). At baseline and month 6, participants who completed study data were entered to win a $100 grocery gift card.

#### Barriers to Care

Other outcomes included barriers to care and survey data (i.e., participant satisfaction, mental relaxation). Barriers to care data were collected for 12 months, obtained from the CHW-participant calling logs and chart review, and grouped into three categories: obtaining medications, appointment access, and clinic eligibility.

#### Survey Data

Survey data were gathered during group visits at month 6. If participants were not present, CHWs called them to conduct the survey. One survey contained 11 questions (participant satisfaction, quality of life (#1–7); open-ended comments (#8–11)) and utilized our feasibility study survey as a template (Appendix). Another survey, Use of Mental Stress Management/Relaxation Techniques, evaluated open-ended type and weekly quantity of relaxation participants experienced.^28^

### Statistical Analyses

Investigators derived the sample size calculation from prior, high-quality group care diabetes studies that evaluated change of HbA1c as a primary outcome.^29–31^ Investigators used the Morris equation^32^ to calculate an estimated effect size (*d*) based on the mean pre-post change in the intervention arm minus the mean pre-post change in the control group, divided by the pooled pre-test standard deviation. Using mean HbA1c data from Sadur et al.,^30^ intervention (pre: 9.48% (SD + 1.9)/post: 8.18% (SD + 0.9)) and control (pre: 9.55% (SD + 1.9)/post: 9.33 (SD + 0.9)), investigators generated an estimated effect size dppc2 of − 0.57. Our targeted sample size and power estimates were calculated using G*Power 3.129.^33^ Based on these data, investigators determined that a total sample size of 78 at the end of the trial was needed, assuming nominal values for the type I and II error rates (i.e., 5% and 20%, respectively, two-tailed) to provide sufficient power to detect a clinically significant HbA1c change.^27, 34^ Assuming a 15% 6-month attrition rate for this population,^10^ a total sample size of *N* = 88–90 was needed for the primary study endpoint. Gpower, *F* test, and ANOVA repeated measures between factors used alpha < 0.05 and power = 0.8.

Continuous variables were assessed for normality and investigators utilized nonparametric tests for nonnormally distributed variables. Investigators used sequential regression and multiple imputation using chained equations (MICE) to handle incomplete data,^35, 36^ growth percentile approach to identify and remove outlier cases from the analysis,^37^ and generalized estimating equations for dichotomized repeated measures (i.e., ADA standards of care).^38^ Linear mixed models were used to evaluate the treatment effect on HbA1c, blood pressure, BMI, and weight change from baseline to month 6. Barriers to care were summated and recorded as percentile. Survey data were evaluated by gathering an item mean score. Investigators defined acceptability as high (3.5–4/4 or 8–10/10), moderate (2–3.4/4 or 4–7/10), and low (1–1.9/4 or 1–3/10) and totalled the items to create an overall score.^39^ If an item was omitted from a survey, the variable was excluded from analysis.

## RESULTS

**Figure 1:**
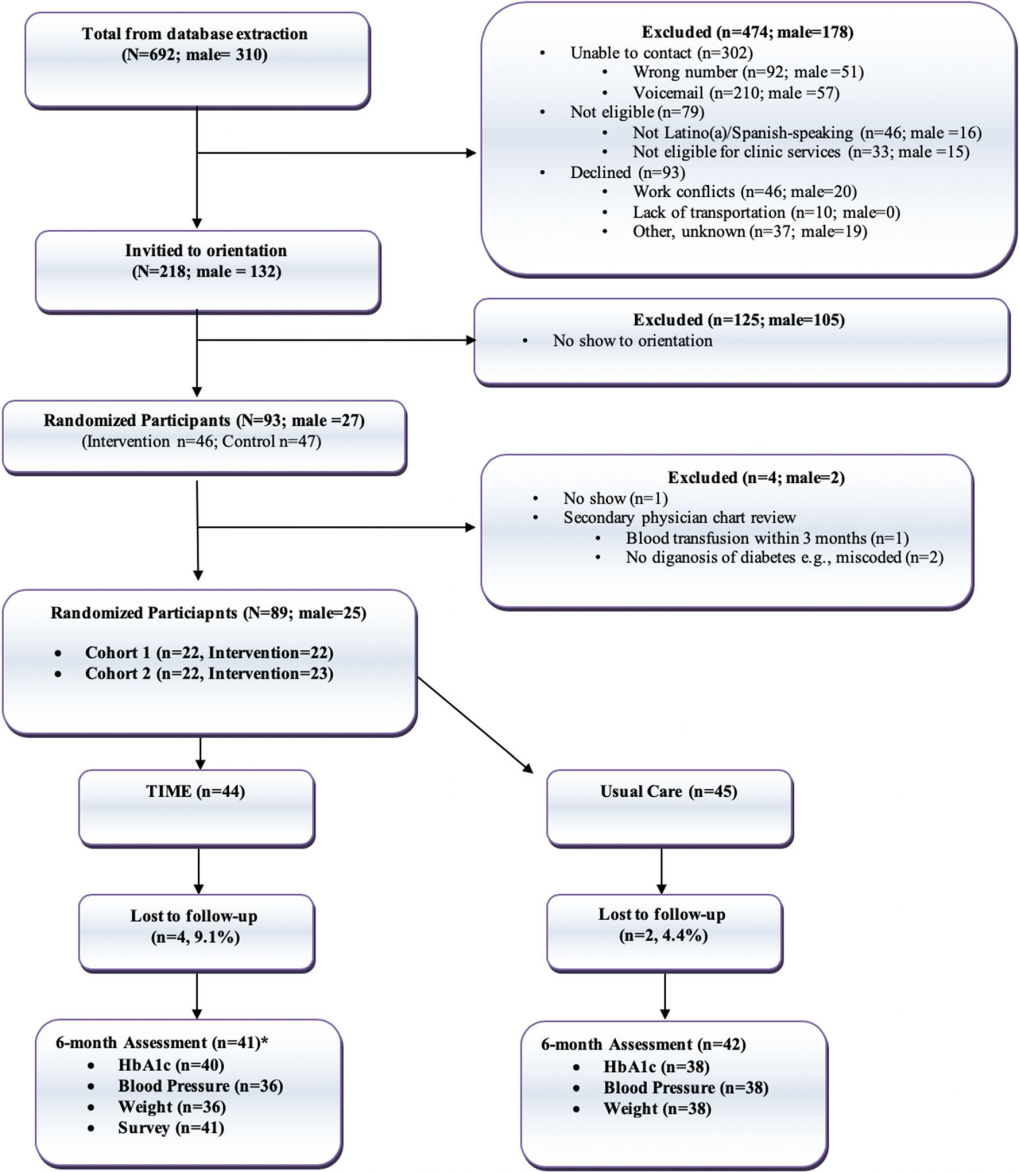
CONSORT diagram of low-income Latino(a)s participating in the TIME (*T*elehealth-supported, *I*ntegrated care with Community Health Workers, and *ME*dication-access) program (intervention) versus usual care (control). The asterisk refers to study attrition defined as inability for study team to contact pariticipant. Several participants could be contacted by the study team but did not show for clinical data or respond within the month 6 timeframe.

The CONSORT diagram (Fig. 1) demonstrates the combined flow of cohorts 1 and 2 from database extraction to study entry. Study staff called 692 (cohort 1: *n* = 352) individuals. There were more females in the database (55.3%) and males had a higher (79.5%) no-show rate at orientation. A total of 89 total participants (intervention = 44) entered the study. Participant demographics are outlined in Table 2. There were no significant differences between groups in age, sex, work history, HbA1c, cholesterol, blood pressure, BMI, or weight. Most participants worked in domestic (*n* = 38) or maintenance/construction (*n* = 22) employments.

**Table 2:** Baseline Demographics and Clinical Information by Study Arm (*N* = 89; Intervention = 44)

Variable	TIME (*n* = 44), *n* (%)	Control (*n* = 45), *n* (%)	*p* value
Sex (female)	34 (77.27)	30 (66.67)	0.271
Work			0.075
Domestic	15 (34.09)	23 (51.11)	
Maintenance, construction	9 (20.46)	13 (28.89)	
Unemployed, unknown	9 (13.33)	6 (20.46)	
Food service	5 (11.36)	2 (4.44)	
Other (retired, office)	6 (13.64)	1 (2.22)	
Variable	TIME (*n* = 44); mean (+ SD)	Control (*n* = 45); mean (+ SD)	*p* value
Age (years)	55.99 (7.12)	53.86 (9.07)	0.220
Diabetes diagnosis (years)	14.28 (9.09)	11.38 (8.56)	0.110
Hemoglobin A1c (%)	9.02 (1.98)	8.71 (2.34)	0.503
Uncontrolled*	9.78 (1.79)	9.76 (2.06)	0.968
Total cholesterol (mg/dL)	180.66 (42.56)	182.47 (44.54)	0.845
HDL cholesterol (mg/dL)	48.02 (10.63)	47.84 (12.85)	0.943
LDL cholesterol (mg/dL)	99.35 (37.02)	101.84 (38.12)	0.758
Triglycerides (mg/dL)	170.02 (76.52)	196.91 (111.31)	0.187
Systolic blood pressure (mmHg)	131.64 (16.22)	130.73 (13.64)	0.777
Diastolic blood pressure (mmHg)	76.41 (7.70)	75.80 (7.99)	0.715
Body mass index (kg/m^2^)	32.59 (6.30)	34.56 (8.22)	0.208
Weight (kg)	80.36 (20.20)	85.75 (22.85)	0.242

*TIME T*elehealth-supported, *I*ntegrated care with Community Health Workers, and *ME*dication-access (intervention)

*Included those who needed > 0.5% HbA1c reduction to achieve target levels (baseline > 7.4% (> 7.9% if > 65 years old))

There was a slight (61.4%) preference for phone calls versus text message for CHW-participant communication. CHWs were usually successful contacting participants (median 85.0%, range 59.1%–100%), and most (83.3%) utilized both phone and text modalities. Group visit attendance averaged 66.7% (84.1% > 50% show). Study attrition was 6.7% (*n* = 4 intervention, *n* = 2 control).

### Clinical Outcomes

**Figure 2:**
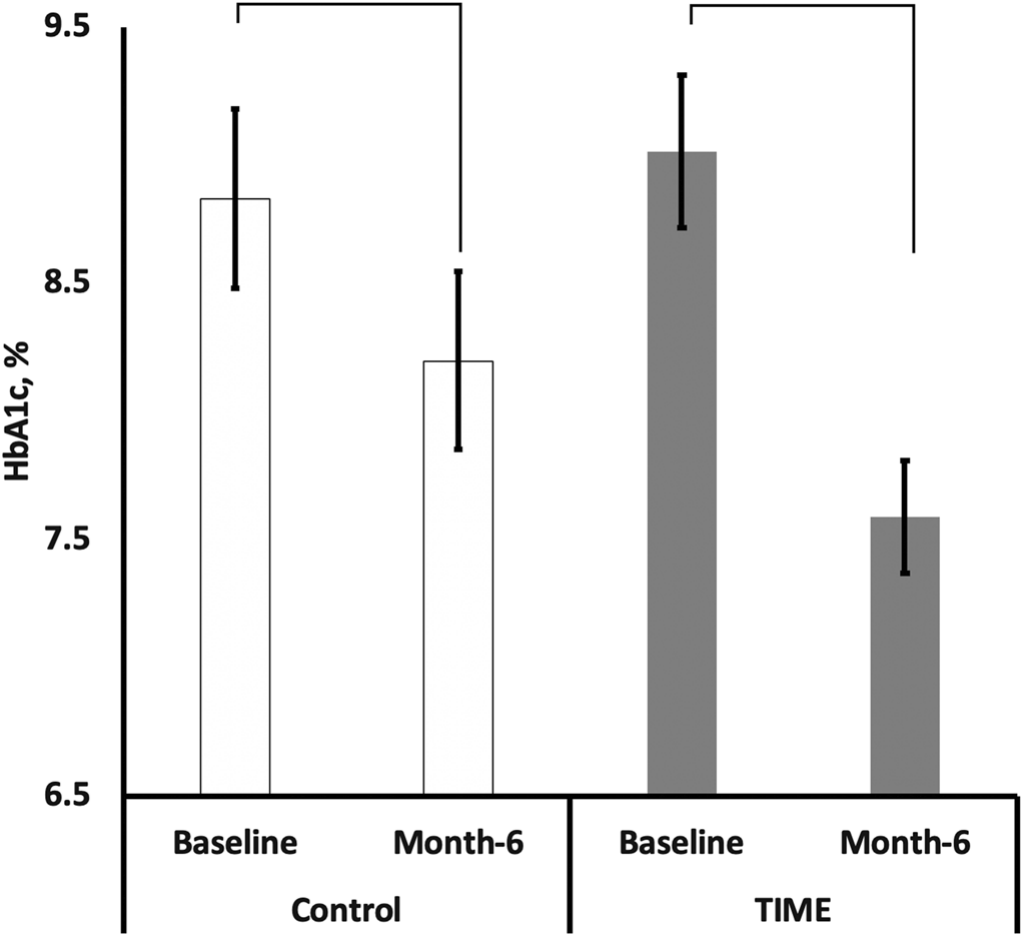
Comparison of HbA1c baseline to month 6 changes by study arm (*N* = 89; TIME (intervention) *n* = 44). There were significant differences between group change of HbA1c from baseline to month 6 (*p* = 0.002). Within group changes were only significant for TIME participants (intervention: *p* < 0.001 (Cohen’s *d* effect size (*d*) = 0.79), control: *p* = 0.356, *d* = 0.20). TIME *T*elehealth-supported, *I*ntegrated care with Community Health Workers, and *ME*dication-access (intervention).

**Figure 3:**
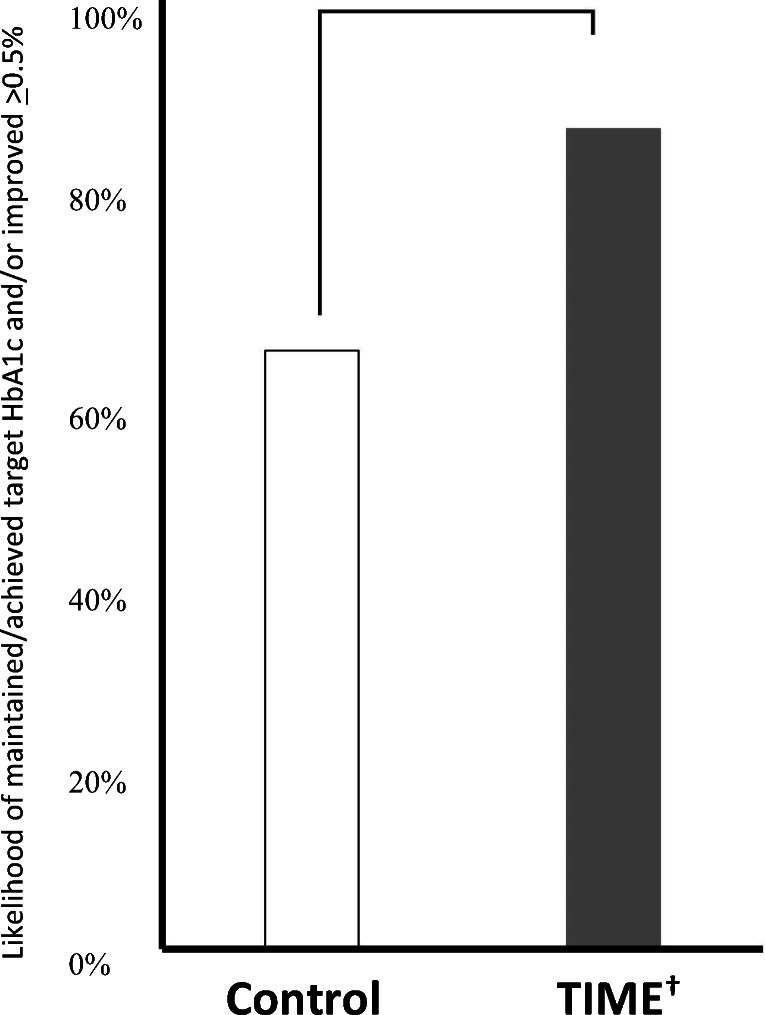
Comparison of *responders* (responder) by study arm (*N* = 89: intervention *n* = 44). There were significant differences (*p* = 0.015) between groups from baseline to month 6. Maintained/achieved target HbA1c (< 7.0% (< 7.5% if > 65 years old)) by 6 months and/or improved HbA1c > 0.5% if baseline HbA1c > 7.4% (> 7.9% if > 65 years old).^3^ The dagger refers to TIME—*T*elehealth-supported, *I*ntegrated care with Community Health Workers, and *ME*dication-access (intervention).

Figures 2 and 3 and Table 3 illustrate the subsequent clinical outcomes. Between-group comparisons showed that TIME compared to control participants had significant HbA1c baseline to month 6 changes (all: − 1.43% vs. − 0.45%, respectively, *p* = 0.002; uncontrolled: − 1.93% vs. − 0.62%, respectively, *p* = 0.007), more *responders* (*p* = 0.015, OR = 2.33; 95% CI 1.21, 9.14), and a greater number who achieved > 0.5% HbA1c reductions (88.57% vs. 43.75%, respectively; *p* < 0.001). Though not significant, fewer TIME participants started at target HbA1c yet groups ended with similar numbers (*p* = 0.143).

**Table 3:** Baseline to Month 6 Clinical Changes by Study Arm (*N* = 89; Intervention = 44)

	TIME (intervention)		Usual care (control)		
	Baseline SD or (%)	Six months SD or (%)	Baseline SD or (%)	6 months SD or (%)	*p* value*
HbA1c (%)					
Uncontrolled^†^	9.78 ± 1.79	7.85 ± 1.59	9.76 ± 2.06	9.14 ± 2.42	0.007
All participants	9.02 ± 1.98	7.59 ± 1.46	8.71 ± 2.34	8.26 ± 2.32	0.002
HbA1c target^**‡**^	6 (13.64)	18 (40.91)	12 (27.27)	19 (43.18)	0.143
HbA1c reduction > 0.5%^†^	n/a	31 (88.57)	n/a	14 (43.75)	< 0.001
Blood pressure (mmHg)					
Systolic	131.64 ± 16.22	124.75 ± 13.08	130.73 ± 13.64	130.76 ± 14.55	0.023
Diastolic	76.41 ± 7.70	73.05 ± 7.49	75.80 ± 7.99	76.00 ± 9.96	0.046
Body mass index (kg/m^2^)					
All participants	32.59 ± 6.30	32.06 ± 6.59	34.56 ± 8.22	33.67 ± 8.46	0.684
Controlled baseline HbA1c	32.84 ± 5.43	31.14 ± 7.26	34.85 ± 7.22	33.99 ± 7.91	0.691
Weight (kg)					
All participants	80.36 ± 20.20	78.72 ± 19.36	85.75 ± 22.85	83.60 ± 23.75	0.823
Controlled baseline HbA1c	77.96 ± 13.52	73.83 ± 17.29	81.74 ± 15.63	79.63 ± 16.49	0.691
Preventive care^**§**^					
B12 screening	0 (0.0)	43 (97.72)	0 (0.0)	0 (0.0)	< 0.001
Statin therapy	23 (52.27)	42 (95.46)	16 (35.56)	22 (48.89)	< 0.001
Comprehensive foot exam	8 (18.20)	44 (1.00)	9 (20.00)	20 (44.40)	< 0.001
Influenza vaccination	10 (22.73)	37 (84.09)	6 (13.33)	12 (26.67)	< 0.001
Pneumococcal vaccination	16 (36.36)	42 (95.50)	10 (22.22)	13 (28.89)	< 0.001
Retinal eye exam	2 (4.50)	44 (1.00)	7 (15.60)	16 (35.56)	< 0.001
Urine microalbumin	4 (9.10)	44 (1.00)	9 (20.00)	17 (37.78)	< 0.001

*TIME T*elehealth-supported, *I*ntegrated care with Community Health Workers, and *ME*dication-access (intervention)

*Reflects baseline to month 6 group comparisons except HbA1c reduction variable, which was month 6 group comparisons

^†^Included those who needed > 0.5% HbA1c reduction to achieve target levels (baseline > 7.4% (> 7.9% if > 65 years old))^3^

^**‡**^Defined as HbA1c < 7.0% (< 7.5% if > 65 years old)

^**§**^Guideline adherence as per American Diabetes Association.^3^ Data were gathered by chart review at the following timepoints: baseline/month 6 (appropriately dosed statin; B12 screening); baseline/month 6 and 1 year prior for each timepoint (annual screenings—retinal, foot, urine, influenza vaccination); baseline/month 6 and time prior as dictated per ADA guidelines (pneumococcal vaccination)^3^

Within-group comparisons also revealed substantially greater effect sizes related to HbA1c improvements in TIME (*p* < 0.001, *d* = 0.79) but not the control arm (*p* = 0.356, *d* = 0.20). TIME compared to control participants resulted in greater systolic (*p* = 0.023) and diastolic (*p* = 0.046) blood pressure improvements and more achieving the seven guideline concordance measures (*p* < 0.001). There were no significant differences in BMI or weight changes between groups. TIME individuals compared to control participants with baseline HbA1c < 7% lost more weight, though not significant (*p* = 0.691) (Table 3).

### Barriers to Care

A total of 54.6% (*n* = 24) of TIME participants reported > 1 barrier to care, of which 62.5% (*n* = 15) experienced > 2. Of these individuals, most (91.7%) experienced barriers related to medications (36.4% incorrect amount/none given, 36.4% lost eligibility to high-cost medications, 27.2% both) and some regarding clinic eligibility (16.7%) and appointment access (8.3%). The majority (87.5%) of these barriers were identified by CHW-participant interaction and the remaining by a study physician.

### Survey Data

TIME participants recorded high satisfaction levels (3.8/4.0 + 0.5). Most agreed that the program met their needs (3.8/4.0 + 0.4), CHW-participant interaction was beneficial (9.8/10 + 0.7), their health was better (9.7/10 + 0.7), and they would come in the future and recommend the class (3.8/4.0 + 0.5, 9.9/10 + 0.4; respectively). TIME participants liked the education, individualized attention, staff, and suggested more than 6 months duration. The most common strategies for mental relaxation (mean two times/week) included exercise (28.2%), spirituality and rest (23.1% each), music (10.3%), and socialization (5.1%).

## DISCUSSION

Diabetes is an epidemic that continues to increase, requiring innovative measures to prevent further burden to vulnerable populations. This study validated that participants in TIME achieved superior glycemic control, blood pressure, and ADA guideline adherence compared to control individuals. Rationale for these findings are an integrated, multifaceted program including the combination of mHealth and CHWs, which is a novel approach to care with promising results that show efficacy.

Compared to our feasibility study, this investigation resulted in superior glycemic and blood pressure outcomes.^6^ Overall, diabetes group visits and CHW interventions have shown effective HbA1c reductions (− 0.46%, − 0.71%, respectively)^8, 10, 11^ but less than those of the current study. This investigation transitioned from quarterly, in-person CHW training in our feasibility study to weekly, telehealth support. Also, this study strongly emphasized low-cost medications and combined CHWs and mHealth, which to our knowledge has not been published before in diabetes clinical trials. These factors likely contributed to the stronger findings from this study compared to our feasibility study and other investigations.

Telehealth (mHealth, video conferencing) was an important part of TIME. mHealth improved communication and connected CHWs and participants to the healthcare system,^5^ whereas video conferencing enhanced consistent training and support.^15^ These modalities likely greatly enhanced patient outcomes by assisting in high (87.5%) CHW ability to identify barriers to care, low attrition, physician communication, and, thereby, improved glycemic control. These findings are consistent with a large body of literature that has shown improved support and communication associated with telehealth.^5, 40^

Notably, the most common barrier to care related to medications and, therefore, the emphasis on low-cost hypoglycemic agents was critical. A study physician prescribed and maximized the most affordable ($4–$6/month) oral medications available at major pharmaceutical retailers^3, 4, 24^ and high-cost agents including insulin were initiated when inexpensive options had been exhausted. Participants taking high-cost medications upon study entry were continued if they remained effective and accessible. CHWs facilitated medication access by alerting a study physician when participants experienced barriers. Prior studies have agreed upon the complexity of medication adherence, noting cost as a major contributor.^4, 24^

While many individuals experienced medication access barriers, others oftentimes were nonadherent due to fear, forgetfulness, and misunderstanding.^41, 42^ This is a complex topic where integrated diabetes care with group visits was important. For example, during the large groups, CHWs emphasized nonadherence issues and subsequent breakout sessions allowed opportunities to discuss individual concerns, address questions, and provide collaboration. CHW-participant mHealth communication provided further education, feedback, and reminders.

Investigators included individuals with baseline-controlled HbA1c levels for two major reasons. HbA1c variability disproportionately affects low-income populations,^43, 44^ and, therefore, controlled individuals may have had substantially different glycemic levels in recent months. HbA1c variability has severe consequences including cardiovascular events, microvascular complications, and all-cause mortality.^43, 44^ Additionally, though there are strong recommendations for diabetes prevention,^3, 45^ most programs exclude those who are controlled. Weight management is particularly important for these individuals;^3^ though not significant, TIME participants who were controlled at baseline lost more weight than the control arm (Table 3). This finding may have been significant with more participants in this subgroup.

Our study is limited by time and a moderate sample size that is majority female, although most diabetes group visit investigations are less than a year, have high attrition, and are minority male.^6, 10–12^ Results are also limited to Spanish-speaking individuals at a nonprofit clinic. Though multifaceted interventions limit deciphering the most efficacious variable, these strategies have been found valuable for the complicated nature of diabetes.^46^ Long-term analyses including the role of behavioral health are difficult to assess in 6 months. Prior investigators found short-term success but failed to demonstrate sustainability.^47^ In the current study, weight-neutral outcomes may also relate to the addition of hypoglycemic medications associated with weight gain.^48^

Several areas of future directions are warranted. Further variable and cost analysis of TIME’s multifaceted approach to diabetes care using multiphase optimization strategies or step-wedge designs are needed. Outcome sustainability after participants re-enter clinic are also warranted; observations suggest no clinically significant HbA1c change at > 12 months for TIME participants. However, individual HbA1c trends demonstrate minimal improvement and possibly significant deterioration, which may be medication access related. Future studies should address clinic-wide strategies for sustainable medication access in low-income settings.

## CONCLUSIONS

This study demonstrates the integral role of CHWs in a multifaceted diabetes program. CHWs are uniquely positioned to recognize medication barriers that would likely have otherwise been unidentified. Telehealth played a key part in supporting the program for both CHWs and participants. To increase the likelihood of sustainability, longitudinal evaluations of TIME are needed to evaluate the ability to implement the program in multiple sites.

## Electronic supplementary material

ESM 1 (DOCX 16 kb)
