# Supplementary material for: A Telehealth-supported, Integrated care with CHWs, and MEdication-access (TIME) Program for Diabetes Improves HbA1c: a Randomized Clinical Trial
Source: J Gen Intern Med. 2020 Jul 22;36(2):455–63. doi: 10.1007/s11606-020-06017-4 (PMC7878600; doi:10.1007/s11606-020-06017-4)
Supplement: Supplementary file 1 — (DOCX 16 kb) [file 11606_2020_6017_MOESM1_ESM.docx]

**APPENDIX**

| Participant exit survey | ☹ ☺ | | | |
| --- | --- | --- | --- | --- |
| Did the diabetes classes meet your needs? | None | Few | Most | Almost All |
| Were you satisfied with the diabetes classes? | Very dissatisfied | Indifferent | Mostly satisfied | Very satisfied |
| Would you come back for these classes? | No | No, I don’t think so | Yes, I think so | Yes, definitely |
|  | | | | |
| On the scale of 1 to 10 (1 is do not agree, 5 is somewhat agree, 10 is definitely agree), please rank: | | | | |
|  | ☹ ☺ | | | |
| Calls or texts from the Community Health Workers were helpful | 1 2 3 4 5 6 7 8 9 10 | | | |
| Having Community Health Workers as part of your healthcare team was helpful | 1 2 3 4 5 6 7 8 9 10 | | | |
| I would recommend these classes to a friend or family member | 1 2 3 4 5 6 7 8 9 10 | | | |
| My quality of life (comfort or happiness) is better because of these classes | 1 2 3 4 5 6 7 8 9 10 | | | |
|  | | | | |
| What did you like about the diabetes classes? | | | | |
| What would you want to change about the diabetes classes? | | | | |
| Comments/Suggestions:  Thank you!! | | | | |
